# Electric Field Improvement for High-Voltage Bushings

**DOI:** 10.3390/polym15010040

**Published:** 2022-12-22

**Authors:** Li Li, Qi Li, Shuxin Xu, Rui Liu, Manling Dong, Si Ying, Jieyuan Tian, Wanpeng Xin, Manu Haddad, Xingliang Jiang

**Affiliations:** 1 Electric Power Research Institute of Guangdong Power Grid Co., Ltd., Guangzhou 510080, China; 2 State Key Laboratory of Power Transmission Equipment & System Security and New Technology, College of Electrical Engineering, Chongqing University, Chongqing 400044, China; 3 State Grid Henan Province Electric Power Scientific Research Institute, Zhengzhou 450052, China; 4 China Electric Power Research Institute, Wuhan 100192, China; 5 Bushing (Beijing) HV Electric Co., Ltd., Beijing 100000, China; 6 Advanced High Voltage Engineering (AHIVE) Research Centre, School of Engineering, Cardiff University, Cardiff CF24 3AA, UK

**Keywords:** high-voltage bushing, resin-impregnated paper bushing, electromagnetic computation, charge simulation method, CSM, finite element method, FEM, electric field calculation

## Abstract

Resin-impregnated paper (RIP) bushing has gained significant interest due to its extended application in Extra High Voltage (EHV) and Ultra High Voltage (UHV) electricity transmission systems. However, the design criterion of its overall structure, the geometry parameters of the condenser layers, and stress release devices, etc., are still not fully understood. This article proposes a unique electric field optimization technique to integrate both the analytical and the numerical methods. The charge simulation method (CSM) is employed to create the overall equipotential surface, within which the finite element analysis (FEA) is adapted to study the localized field enhancement effects, taking into consideration the multi-physics coupled fields. A case study is performed on an actual UHV bushing. The results are compared to the traditional methods, to demonstrate the benefit of the hybrid method.

## 1. Introduction

Bushings play a critical role in the provision of electrical insulation and mechanical support in many types of power equipment, such as transformers, reactors circuit breakers, etc. It is believed that bushings are the key technical limitation in Ultra High Voltage (UHV) electricity transmission design, especially when the voltage increases above 500 kV. Design improvement of UHV bushings is therefore significant for increasing the voltage levels of modern power systems. However, the rapid increase in voltage levels within modern power systems has imposed more and more challenges on the reliability of high-voltage bushings. There are few studies focused on the key design parameters of bushings, such as the overall structure of bushings, the geometric parameters of condenser layers, and stress-relief devices. Methods from the existing literature on electric field calculations are diverse, either through numerical or theoretical routes.

Sun calculated the field-strength distribution and the maximum value point of the field strength of the casing under different structural sizes in 2001. According to the calculation results, the optimized design size and some improvement measures were provided for engineering practice, which were verified by experiments [1]. In 2003, in order to improve the uneven and over-concentrated electric field distribution of stress-controlled bushings, Yang adopted the combined structure of nonlinear resistance material and a capacitor band with a high dielectric constant, and simulated its equivalent circuit by PSPICE. Three samples were made according to different treatment methods and a partial discharge test was carried out [2]. Jiang carried out a 2D electric field analysis on the composite casing, made of epoxy resin and high-temperature vulcanized silicone rubber, with ANSYS analysis software in 2004, and discovered the method of using the aspect ratio of a 1:3~1:4 field to deal with the far-field problem in an electric field with high aspect ratio. The calculation showed that the maximum field strength can be reduced by 33.5% by brushing semiconductor paint on the joint between the casing and flange [3]. Xu used finite element method software to calculate the field-strength distribution and potential distribution inside and outside the porcelain bushing with or without grounding internal shielding in 2007. The influence of the position and size of the grounding inner shield and the electric field distribution was analyzed, and the structure of the grounding inner shield was optimized, which provided a theoretical basis for the design of the 126 kV SF6 bushing grounding inner shield [4]. In 2008, Di applied ANSYS finite element analysis software to analyze the electric field distribution of non-capacitive composite bushings made of epoxy resin and high-temperature vulcanized silicone rubber. The results of improving the uniformity of electric field distribution and meeting engineering needs were obtained [5].

According to the preliminary design structure of the circuit breaker outlet bushing, Liu analyzed the insulation performance based on the numerical solution of the electric field. An axisymmetric electric field mathematical model of the circuit breaker outlet bushing was established, and electric field numerical simulation and visual processing of the outlet bushing were carried out [6]. Zhong calculated and analyzed the design and development of 1100 kV composite silicone rubber bushings by using ANSYS analysis software. According to the calculation and analysis results, the UHV silicone rubber composite bushing met the needs of the project as designed, and the optimized bushing structure was obtained [7]. In order to prevent rain flash accidents, Chen formulated a scheme of adding an auxiliary umbrella skirt to the high-voltage bushing of a 500 kV main transformer in 2012, and conducted a test analysis. The results showed that the bushing could effectively isolate rainwater and arc after installation of the auxiliary umbrella skirt, and its withstand voltage was significantly improved. According to the test results, the installation requirements, acceptance requirements and maintenance requirements for the installation of auxiliary umbrella skirts on the high-voltage bushings of 500 kV main transformers were put forward [8].

Yu used QUICKFIELD software (Tera Analysis Ltd. Svendborg, Denmark.) to analyze the electric field of the bushing, and optimized its structure [9]; Wang used CAD three-dimensional modeling software (Autodesk. San Rafael, California, USA) to realize the valve A step-by-step sub-model solution method proposed for the fine modeling of the interior of the hall [10]. In 2015, Wang used finite element analysis software, ANSYS (ANSYS, Inc. Southpointe 2600 ANSYS Drive Canonsburg, PA 15317 USA), to establish a three-dimensional finite element model of key equipment, such as double valve towers in the valve hall, in order to study the electric field distribution of each piece of equipment and voltage-equalizing the shielding device in the UHV ± 1100 kV valve hall. The state field analysis method was used to calculate the overall potential and electric field distribution of the valve hall in one power–frequency cycle, and to obtain the surface electric field strength of the pressure-equalizing shielding device of each piece of equipment in the valve hall [11].

Cao studied the structural design of DC bushing iron core in 2013, and the electrode plate of capacitor iron core was adjusted to control the current distribution through theoretical derivation [12]. Zhu simulated and analyzed the electric field distribution under different capacitor core spacing and thickness using MATLAB (MathWorks. Natick, Massachusetts, USA) [13]. Zhang studied UHV DC through wall bushings from 2013 to 2015. When calculating the electric field strength of the bushing, the maximum voltage was applied to the inner conductor, and the voltage of the flange and valve hall wall was zero. The rest adopted floating potential [14,15,16]. Zhao studied the influence of EHV Transformers with grading ball insulation structure bushings, on electric field distribution in 2016, established a simulation model of a transformer high-voltage outgoing line system, and applied electro V6.2 (Electro sensors, Inc. NASDAQ). The software carries out the finite element analysis of the two-dimensional electric field, calculates and analyzes the safety margin, and discusses the influence of the change in arc radius of the voltage-equalizing ball on the field strength and safety margin, so as to optimize the insulation structure of the voltage-equalizing ball and improve the electrical and safety performance of the transformer [17]. Wang also conducted research on electric field calculation in 2017, but the analysis method of three-dimensional electromagnetic-fluid–thermal coupling was mainly considered in the calculation of electric field distribution, focusing on the coupling effect of the temperature field on the electric field calculation [18,19]. In 2018, Yang conducted a three-dimensional simulation study on the electric field distribution of 800 kV converter transformer bushings. The temperature rise changed the resistivity of the insulating medium, thus affecting the field strength distribution and distorting the field strength on the surface of the silicone rubber umbrella skirt of the insulating jacket. Due to the effect of the temperature gradient, the resistivity of the insulating medium inside the casing was affected, the distribution of the electric field inside the casing was changed, and the maximum value of the field strength was increased. The maximum value of the field strength at the flange of the UHVDC casing—which is more prone to insulation damage, due to the weakness of its surface electric field strength [20]—was increased. The method used was the same as above. Wang simulated electric field distribution by using a guide rod with high potential and the outermost and flange grounding in a study of the influence of water distribution on electric field distribution in 2019. Except for the zero electrode and the high-voltage electrode, the remaining electrodes in the bushing had floating potential [21]. Chen adopted the same method in assessing the influence of material volume conductivity on bushing electric field distribution under DC electrothermal coupling stress in 2020 [22].

Improving electric field distribution has always been the key to effectively optimizing bushing structure. Jin optimized electric field distribution by changing the internal shielding structure [23]. Hesamzadeh used a genetic algorithm to adjust the geometry of the electrode plate and foil to determine the best design sketch of a capacitor grading sleeve, but this scheme could not effectively reflect the electric field on the edge of the electrode plate [24]. Dai used Infolytica to simulate and optimize the edge design of a bushing plate with a two-dimensional axis-symmetrical model [25].

In this article, a 126 kV transformer bushing (DC) was selected for electric field calculation. Commercial software COMSOL (COMSOL. Stockholm, Sweden) and MATLAB were used to combine analytical and numerical methods. A finite element analysis (FEA) model was established to simulate the electric field distribution of capacitive bushing. The FEA method refers to Li’s paper [26,27]. The surface field strength calculation refers to Li’s paper [28,29]. The results are compared with the traditional method, and a design scheme meeting the insulation conditions is proposed. Results are compared to the traditional methods to demonstrate the benefit of the hybrid method.

## 2. Materials and Methods

### 2.1. Theory

In COMSOL electrostatic interface, the electric field intensity and potential distribution can be calculated for complex geometries such as 2D axis-symmetric or 3D. The other advantage is that all spatial dimensions support the establishment of frequency- and time-domain analysis. Take two-dimensional axisymmetric space condition as an example; the scalar potential is selected as the dependent variable to solve the Gaussian law.

Maxwell equations are solved for electromagnetic field; that is, solve the equations related to the following variables:

(1) $$\left\{\begin{array}{l} \nabla \cdot E=\frac{\rho }{\varepsilon_{0}}\\ \nabla \cdot E=0\\ -\nabla V=0\\ \nabla \times \nabla V=0 \end{array}\right.$$

where *ρ* refers to space charge density, electric field is *E*, potential is *V* and dielectric constant of free space is $\varepsilon_{0}$. The information involved in Maxwell’s electrostatic equation can include the above four formulas. Enter the following equation:

(2) $$-\nabla \cdot \nabla V=\frac{\rho }{\varepsilon_{0}}$$

Due to the nature of FEA, the influence of dielectric material on simulation can be extended beyond the above formula. The induced polarization effect is also considered in this case. Bound and free charges act together. When the bound charge is replaced by an external electric field, pairs of positive and negative charges, namely electric dipoles, will be induced. The polarization effect will change the electric field of the filling material of the model. The calculation process involves the following equations:

(3) $$\left\{\begin{array}{l} \rho_{P}=-\nabla \cdot P\\ \nabla \cdot E=\frac{\rho +\rho_{P}}{\varepsilon_{0}}\\ D=\varepsilon_{0}E+P\\ \nabla \cdot D=\rho \end{array}\right.$$

where the above physical quantities are polarization vector field *P*, polarization charge density $\rho_{P}$, and electric field displacement *D*.

If the condition of non-rotation of the electric field is retained, the following equation can be obtained:

(4) $$-\nabla \cdot \left(\varepsilon_{0}\nabla V-P\right)=\rho$$

The relationship between polarization vector field and electric field is as follows:

(5) $$P=\varepsilon_{0}\chi_{e}E$$

where the proportional constant $\chi_{e}$ is the electric polarizability.

By considering the influence of materials on the results, the electrostatic equation can finally be written as:

(6) $$-\nabla \cdot \left(\varepsilon_{0}\varepsilon_{r}\nabla V\right)=\rho$$

In COMSOL, the simulation can start with the simplest capacitor. Combined with the charge simulation method, the virtual charge used is equivalent to the continuous charge distributed on the dielectric surface. The calculation model of bushing electric field is established by using electrostatic condition. When calculating the capacitance of a cylindrical capacitor, it can be assumed that the charges on the relative parallel plane are, respectively, *±Q*, and the electric field intensity in the period can be obtained according to the Gauss theorem:

(7) $$E=\frac{k}{2\pi \varepsilon_{r}}$$

where *k* is the charge per unit length of the cylindrical capacitor relative to the parallel plane.

The voltage between them is:

(8) $$U=\int_{R1}^{R2}E\cdot dL$$

Simultaneously, the Gauss theorem can be obtained:

(9) $$U=\int_{R1}^{R2}\frac{k\cdot dr}{2\pi \varepsilon_{r}}=\frac{k}{2\pi \varepsilon_{r}}\cdot \ln\frac{R1}{R2}$$

Then, the capacitance of the cylindrical capacitor can be simply expressed as:

(10) $$C=Q/U=\frac{2\pi \varepsilon_{r}L}{\ln\left(R1/R2\right)}$$

**Figure 1:**
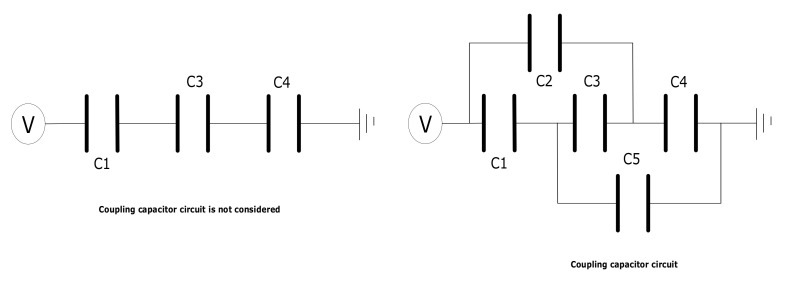
Equivalent circuit diagram of parallel plate capacitors without considering coupling capacitance and considering coupling capacitance.

Integrating the above formula, the screen potential obtained by voltage division is a common method in bushing analysis, but the influence of coupling capacitance is not considered, as shown in Figure 1.

The left side of the figure above is the equivalent circuit diagram of the factory empirical formula, and the potential of each screen can be obtained through programming calculation. On the right is the equivalent circuit, considering the coupling capacitance. In COMSOL, the influence of the coupling capacitance on the overall potential can be considered by setting the suspension potential conditions. When determining the bushing size, such as the screen length, thickness and mutual clearance of bushing capacitor screen, the calculation can be carried out.

### 2.2. Geometric Modeling and Materials

**Figure 2:**
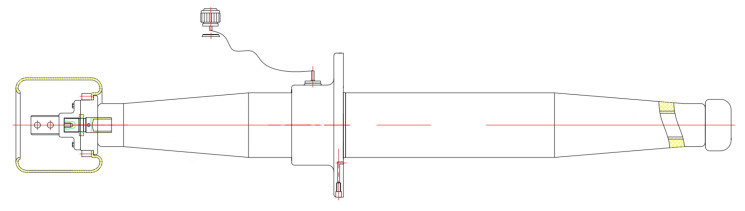
Overall two-dimensional structure diagram of 126 kV highvoltage bushing geometric modeling.

The established bushing model is divided into the following parts: guide rod, capacitor screen, oil pipeline, expansion sleeve, flange, etc. The overall two-dimensional structure of 126 kV high-voltage bushing geometric modeling is shown in Figure 2.

The overall structure of the bushing is shown in the figure, and the modeling mainly focuses on the internal insulation.

The upper side outside the bushing is air, the lower side is transformer oil, the metal parts such as the guide rod are made of aluminum and the filling part around the capacitor screen is epoxy resin. The electrical permittivities are: 1, 2.2, 0 and 5.5, respectively. However, metal did not take part in computer calculation of the electric field.

### 2.3. Boundary Conditions and Meshing

There may be obvious edge field in the simulated bushing, so the air area is also included in the model. The infinite element domain in COMSOL is used to expand and perfect the model field. The dispersion field strength can be extended infinitely, and the strength is inversely proportional to the third power of distance, in theory. For the part where the field strength decreases rapidly, there is no need to worry about the influence of the value on it. In the electrostatic field setting, it is not necessary to solve the specific potential in the capacitor screen, but only the electric field distribution of the dielectric on the oil side and the bushing itself. The grid division method and selected accuracy of the capacitor screen refer to the following papers: [30,31,32,33]. Therefore, the terminal domain feature can be selected in the setting of the physical field interface.

Grid division information is shown in Table 1.

## 3. Results and Discussion

### 3.1. Comparison between Classical Calculation Method and COMSOL Simulation Results

**Figure 3:**
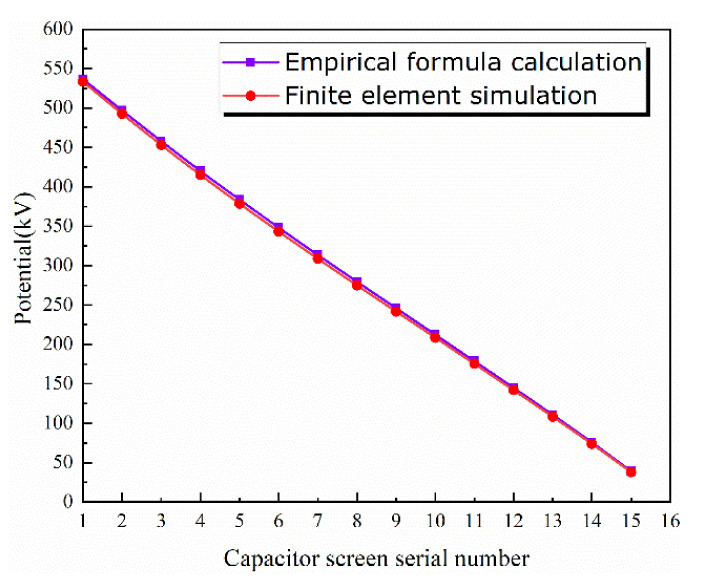
Comparison between empirical formula and finite element calculation results.

**Figure 4:**
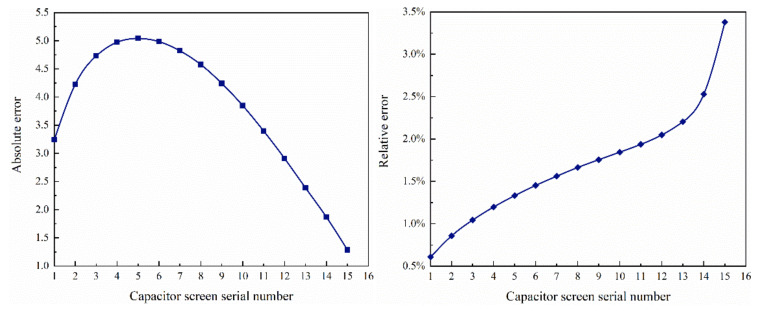
Results for absolute error and relative error.

In the previous section, two different calculation methods were introduced, and their calculation results were compared. The results are shown in Figure 3 and Figure 4.

The true value is the result of the analog value. The absolute error first increases and then decreases, reaching the maximum on the fifth screen. The relative error is basically below 2.5%. The difference between the simulation results of COMSOL and the actual bushing potential is very small, which can ensure the accuracy of the results.

**Figure 5:**
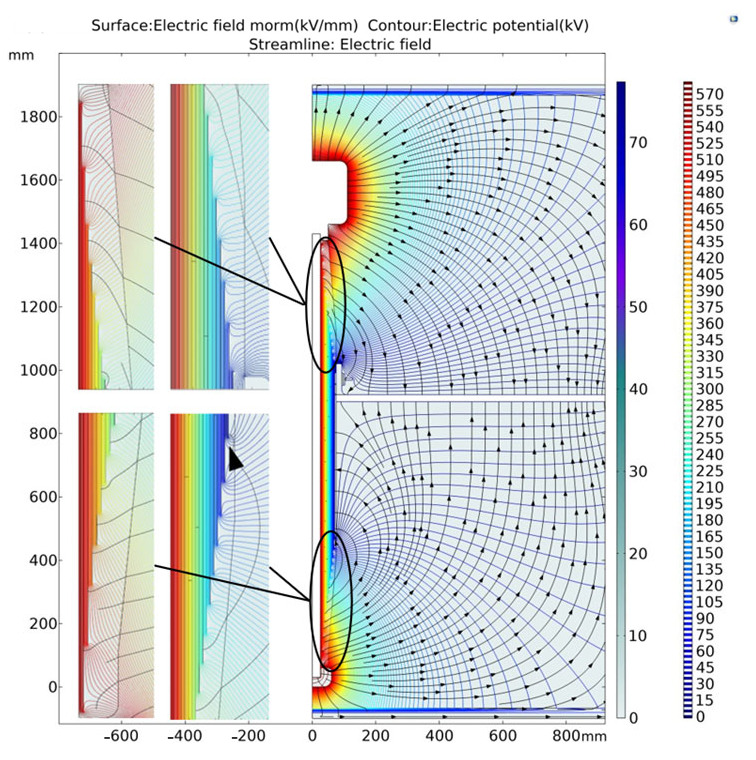
Comprehensive electric field norm, electric potential and electric field.

COMSOL simulation shows the comprehensive electric field norm, electric potential and electric field of 126 kV bushing shown in Figure 5.

From the above figure, we can clearly see the result diagram under the condition of floating potential. The dielectric constant value in the upper left corner is 5.5.

**Figure 6:**
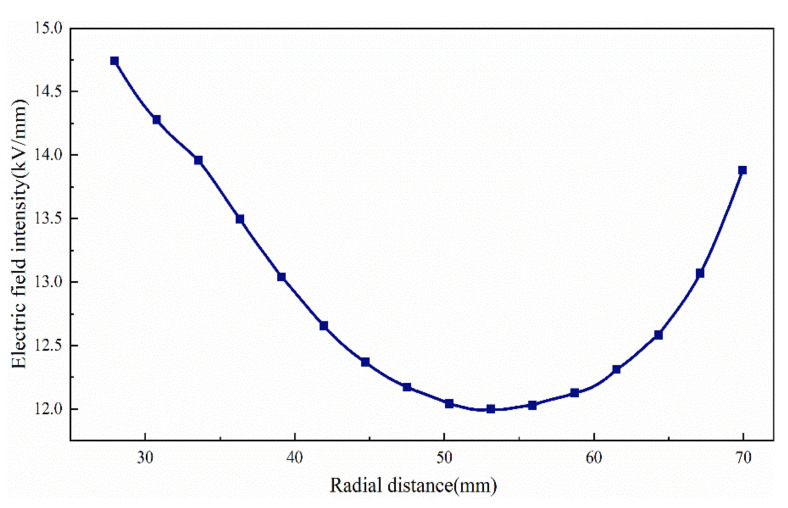
Radial inner field strength of bushing capacitor screen.

The bushing takes the radial inner field strength of the capacitor screen, and the results are shown in Figure 6.

In fact, the distribution of the radial electric field is not uniform, and there is a voltage difference between the maximum and minimum values. In the optimization design, we should consider reducing the difference between them.

### 3.2. Effect of Dielectric Constant of Epoxy Resin on Results

**Figure 7:**
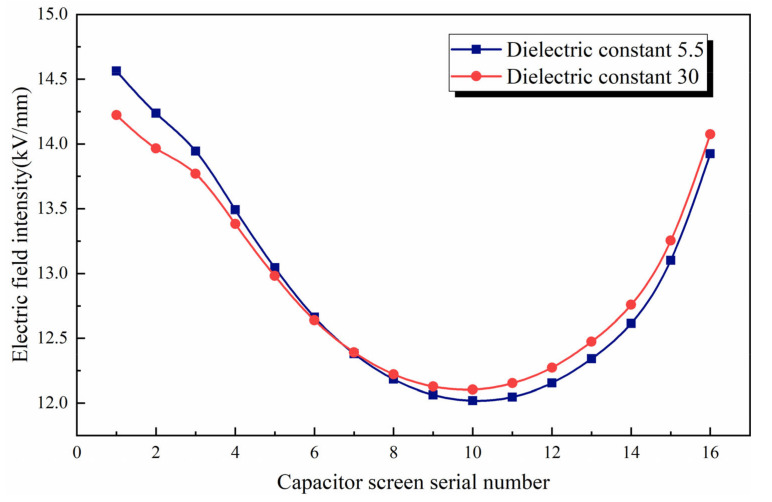
Comparison diagram of comprehensive electric field intensity under different dielectric constant conditions.

Epoxy resin is the filling material inside the bushing and outside the capacitor screen. Under the actual operating conditions, the value of its dielectric constant will change. In particular, material aging of the bushing is caused by moisture, and the insulation performance is affected to a certain extent. In serious cases, there will even be leakage. In COMSOL, the change in the result can be visualized by changing the dielectric constant within a reasonable range. The dielectric constant of epoxy resin is changed from 5.5 to 30 to obtain the radial inner field strength of the bushing capacitor screen, as shown in Figure 7.

It can be seen from the figure that when the dielectric constant of epoxy resin is changed from 5.5 to 30, the comprehensive electric field intensity decreases first and then increases with the increase in the radial distance of the capacitor screen. That is, the increase in dielectric constant of the same material is not friendly to the bushing, and there is a risk of leakage if it exceeds a certain range. However, its ups and downs have indeed flattened.

### 3.3. Optimal Design of Size Structure

We changed the radial distance from the end screen of the bushing capacitor screen to the flange, increased the overall radial length of the bushing, and put forward new thoughts on the actual operation of the bushing under the condition of meeting the insulation requirements.

**Figure 8:**
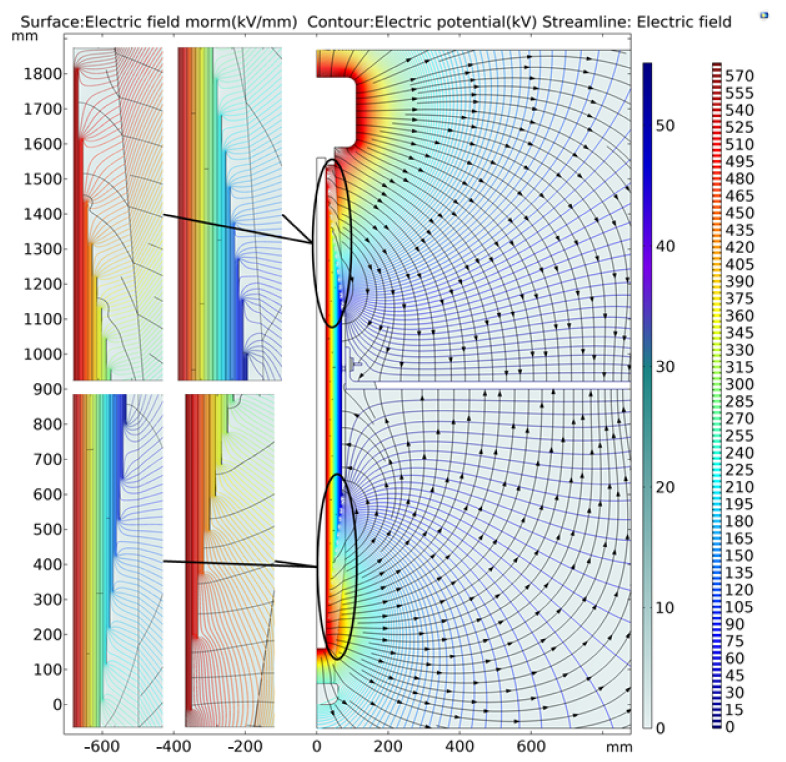
Comprehensive electric field norm, electric potential and electric field.

The radial distance between the end screen of the original capacitor screen and the flange is 430 mm, which is now adjusted to 300 mm for simulation calculation. Additionally, the casing bushing body is increased accordingly. The simulation results of the new design scheme are shown in Figure 8.

**Figure 9:**
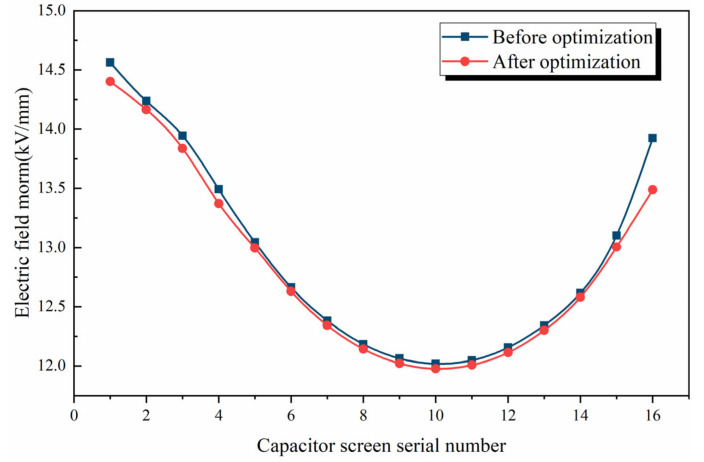
Comparison of electric field intensity inside capacitor screen before and after optimization.

The electric field intensity on the radial inner side of the capacitor screen is compared before and after optimization, as shown in Figure 9.

It can be seen from Figure 9 that the radial distance between the end screen and the flange has an impact. Compared with the original results, the electric field intensity inside the optimized bushing radial capacitor screen shows a downward trend. After optimization, the electric field intensity of the first screen and the last screen of the capacitor screen is significantly reduced, and the fluctuation degree is also slowed down. The adjustment of the design is only a preliminary attempt, and the most obvious result is the decrease in the average electric field intensity.

## 4. Conclusions

In this paper, the finite element analysis model established by the actual bushing analysis was used to study electric field distribution and internal insulation material filling of bushings in a complex multi-physical coupling environment. The results were compared with the classical formula, and problems related to the internal insulating material filled around the capacitor screen were discussed. The advantages of a hybrid method in complex modeling were proved. The following conclusions are drawn:

- By comparing the potential inside the capacitor screen with the traditional design scheme, it is proved that the simulation physical field setting is accurate, has high correlation with the actual parameters, and that the modeling effect is good. The relative error is basically below 2.5%.
- The modeling shows the comprehensive field strength distribution, equipotential line, and radial and axial field strength distribution of the bushing. The electric field between the electrodes of each layer of the bushing capacitor screen is relatively uniform, and there are obvious changes at the electrode plate.
- The dielectric constant of the internal insulating material filled around the bushing capacitor screen will change under the actual operating conditions. Take the normal dielectric constant 5.5 and the aging dielectric constant 30 for analysis and judgment. It can be seen that the influence of the increase in dielectric constant on the electric field strength of the bushing first decreases and then increases. That is, the increase in dielectric constant caused by factors such as humidity and aging will affect the actual operation of the bushing and the risk of leakage.
- The improved optimization design based on the simulation model can improve the electric field distribution and size by increasing the radial length of the bushing on the premise of meeting the insulation requirements. This has practical guiding significance.

## 5. Acknowledgment

This work has been funded by Electric Power Research Institute of Guangdong Power Grid Co., Ltd., Grant Agreement No 0361002022030103KC00068.

## Figures and Tables

**Table 1:** Pollution test results.

Unit Type	Quantity	Grid Parameters	Statistical Information
Triangular element	423,659	Number of units	423,659
Edge element	34,064	Grid area	1,976,000
Vertex element	183	Minimum unit mass	0.06276
		Average unit mass	0.8478

## Data Availability

Not applicable.
